# Clinical Notes from Practice

**Published:** 1891-06

**Authors:** R. C. Hodges

**Affiliations:** Galveston


DANIEL'S

### TEXAS MEDICAL JOURNAL.

A Representative Organ of the Medical Profession, and an Exponent of Rational Medicine; devoted to the Organization, Advancement and Elevation of the Profession in Texas.

PUBLISHED MONTHLY.—SUBSCRIPTION $2.00 A YEAR.

Vol. VI. AUSTIN, JUNE, 1891. No. 12.

### Original Articles.

CONTRIBUTED EXCLUSIVELY TO THIS JOURNAL.

*The Articles in this Department are accepted and published with the understanding that we are not responsible for, nor do we indorse the views and opinions of the writers by so doing.*

---

For Daniel's Texas Medical Journal.

### CLINICAL NOTES.

FROM THE PRACTICE OF R. C. HODGES, M. D., GALVESTON.

CASE I. J. P. B. consulted me for loss of voice. Had been under treatment with sprays and gargles for chronic pharyngitis and laryngitis for nearly two years. Examination of larynx revealed small tumor of left vocal cord, apparently a mucous or fibroid polypus. After a few days of manipulation of the parts with instruments, to render them tolerant, I applied a twenty per cent. solution of cocaine and with Seilers' Laryngeal Guillotine removed the growth successfully. Examination showed it to be a mucous polypus. The result was eminently satisfactory, the voice being fully restored and no recurrence of the growth.

The solution of cocaine used may appear stronger than necessary, but I have found that in operations on the larynx the anæsthetic effect of the ordinary solutions of cocaine, say from five to ten per cent., so rapidly disappears, as to leave very little time for an operation requiring care and skillful manipulation.

Case II. Mr. D. S. consulted me for the same trouble—loss of voice. On examination found large fissure of the epiglottis—the result of ulceration—and in addition stenosis of the larynx.

This condition did not yield to local treatment, and I warned patient to be prepared at any moment for an operation. About two weeks after consulting me he came to my office in the forenoon, about half past eleven, and at twelve o'clock I was summoned to his bedside and found him rapidly succumbing to the impediment to respiration, and proceeded immediately to perform tracheotomy, making one free incision down to the trachea, and second incision opened the trachea and I inserted tube. There was considerable hemorrhage and some blood entered the trachea. The patient was so nearly unconscious that it was with difficulty I could persuade him to use what remaining strength he had in filling his lungs, and in addition applied artificial respiration, holding the tube in place for nearly two hours. Dr. Fisher, who was with me during the operation, and Dr. Sampson present later on, both expressed the opinion that the case was a hopeless one. We, however, persevered in our efforts, and I had the satisfaction, ten days later, of seeing him resume his work in the railroad yards where he had been previously employed. During convalescence there was no rise of temperature, and for several months following the operation the voice was restored and the patient increased twenty pounds in weight.

Five months after the operation the patient, of his own accord and against my protest, removed the tube. In four days the wound healed and a week later the voice had become reduced to a hoarse whisper, and in which condition it now remains. The ulceration of the epiglottis healed nicely under local applications, leaving, however, a large fissure in epiglottis.

The ulceration and stenosis were no doubt due to traumatism, from the history patient gave.

### A GROUP OF EYE CASES.

Case I. Mr. S. consulted me within the past year for a small growth on the conjunctiva of the lower lid. Had been wearing an artificial eye for several years, and suppose that this caused the local trouble.

From the location and character of the growth, I suspected epithelioma, and at all events advised excision of the growth. Operation was made under cocaine, the growth was removed entire, the incision extending well into the healthy tissue.

Microscopical examination of the growth by Dr. George Dock, of Galveston, revealed it to be tuberculous. The patient's own and general family history was most excellent, and thorough examination failed to show any affection of any other organ or part of the body. These cases are very rare, only few of them being on record.

Case II. Mr. B. L. consulted me for suspicious growth of the ocular conjunctiva, but after a thorough examination I was satisfied that I had a true syphilitic papule, a rare location for this lesion. A few days later a syphilitic iritis appeared in the same eye. Both conditions yielded to the local use of atropine and vigorous constitutional treatment; the secondary symptoms of this case were very erratic and the patient could give no history or date of the initial lesion.

Case III. Miss P. consulted me for defective eyesight. Had never been able to see objects distinctly, except close at hand, and was unable to read, but stated that while in stooping posture she could see objects on the floor distinctly. This, I found, was due to a double dislocation of the lens of both eyes, the lens being displaced downward and tilted backwards. The tremulous iris and mobility of the lens in both eyes indicated fluidity of the vitreous in such a degree that I declined to make an operation.

Case IV. Mr. D. consulted me for operation for relief of cataract, very nearly mature, in both eyes; apparently soft cataracts.

I made the needle operation under cocaine and found the cortex soft and readily absorbed, leaving a small hard nucleus. A few months later, in making a corneal flap incision, preparatory to removing the nucleus I found the vitreous fluid and lost so much of it that not only the anterior chamber, but the whole eye collapsed. I immediately removed the speculum, turned patient on his face and kept him there for half an hour. Examination showed the corneal wound closed, the eye ball in proper shape and the anterior chamber filled with air—the nucleus of the cataractous lens lying at the bottom of the deep cone-like anterior chamber, the iris looking directly backward. There was very little pain and no hemorrhage, but within twenty-four hours inflammatory symptoms appeared and severe iritis developed. In four days the large bubble of air had absorbed, the iris had advanced and at the end of three weeks inflammatory symptoms yielded to treatment with atropine and hot water.

I fully expected hemorrhage or detachment of the retina, but neither followed.

The diminution in the size of the cataract and the absorption of the cortical portion of the lens has given patient some vision, and I have since attempted to break up the nucleus by the needle operation. This has not been successful, and I shall shortly make a small corneal incision and introduce a fine hook and attempt to remove the hard nucleus.

This case was particularly interesting to me, as a careful examination before operation failed to show any symptoms of fluid vitreous, and the present condition of the eye, considering the extensive loss of vitreous, is quite remarkable.

Every year adds to my belief that although the eye is one of the most delicate and sensitive structures, it is in some respects one of the most tolerant of any of the parts cf the body.

Case V. Essie T., four years of age, was brought to my office apparently suffering from a simple conjunctivitis. After a careful examination and search I concluded that it could not be due to a foreign body, as I passed a probe behind the upper lid and swept it back and forth several times. But three days later, on raising the upper lid, I saw, presenting, a small black point, and with a pair of iris forceps I seized this, and to my surprise, with gentle traction removed a piece of a twig three-quarters of an inch long and fully one-eighth of an inch in diameter, which undoubtedly had been lodged in the recto-tarsal fold for four weeks The conjunctivitis disappeared without further treatment.

Case VI. J. Nelson,—traumatism,—laceration cornea iris and lens by piece of rock, while blasting. Came to me while inflammation was at its height. At first I feared the eye would have to be eneucleated, but vigorous treatment allayed inflammation. As the eye improved I discovered apparently imbedded in the lens the small triangular piece of rock that had caused the mischief, and proceeded to operate for its removal. The lens was clear to a remarkable extent, the opacity being triangular in shape, with base downward. Made corneal flap and iridectomy downward, lacerated capsule and to my surprise found the foreign body to be the lacerated portions of iris and lymph resulting from the injury, the foreign body having penetrated but did not remain in the eye. I used lens scoop and removed lens, and in a few weeks eye cleared up giving V. $\frac{20}{70}$. I was never so sanguine of anything as of the presence of this foreign body I did not find. Pupil, owing to adhesions, was nearly round, and it would be difficult to detect that an iridectomy had been made.

